# Applications of Blockchain Technology to Higher Education Arena: A Bibliometric Analysis

**DOI:** 10.3390/ejihpe11040101

**Published:** 2021-11-10

**Authors:** Carlos Reis-Marques, Ronnie Figueiredo, Miguel de Castro Neto

**Affiliations:** 1 NOVA Information Management School (NOVA IMS), Universidade Nova de Lisboa, 1070-312 Lisboa, Portugal; crmarques@novaims.unl.pt (C.R.-M.); mneto@novaims.unl.pt (M.d.C.N.); 2 Centre of Applied Research in Management and Economics (CARME), Polytechnic of Leiria, 2411-901 Leiria, Portugal; 3 Research Center in Business Sciences, NECE (UBI), 6200-209 Covilhã, Portugal; 4 Spinner Innovation Centre (SIC), 1600-237 Leiria, Portugal

**Keywords:** blockchain technology, bibliometric studies, disrupt higher education, digital transformation

## Abstract

Research related to blockchain is rapidly gaining importance in the higher education. This opportunity collaborates with a proposal for a review of papers on the main blockchain topic. The bibliometric analysis included 61 peer-reviewed articles published in the Scopus database during the period of 2016 to 2021. This paper offers the identification of gaps in the literature enabling studies on the subject in higher education. The article identifies the main applications of blockchain technology in higher education around the world, as well as suggests future investigations. For further scientific investigation, we propose the operationalization of each of the researched approaches, especially combining the blockchain relationship, artificial intelligence, digital innovation, digital maturity, and customer experience in higher education.

## 1. Introduction

The global evolution has transformed the industrial era into the connected era, with smart products, especially in contemporary organizations [1]. In addition, the advent of the Bitcoin system brought another boom on the Internet, enabling the development of applications in various economic sectors using Blockchain systems [2].

In this context, blockchain is a technology applied in various businesses, originating in cryptocurrency, and developed in higher education [3]. Furthermore, the use of this technology is aimed at cost reduction, information security, and document verification processes [4].

With the growth in the number of international students in the world, and the advance of document forgery, blockchain technology is offered as a secure way to carry out digital transactions [5]. At last, recent studies reveal that there is a growing number of counterfeit educational certificates produced by dishonest candidates for higher education around the world [6].

The use of blockchain can change the way information is exchanged between chain actors as it provides a platform to solve the problem of tracking product information in supply chain management [7]. Today, the great challenge lies in the qualification of the workforce, being applied through digital platforms Lizcano et al. [8].

In addition, the speed of global changes demand a quick adaptation to the new conditions of higher education, creating advances in the application of technologies and innovations in universities [9]. On top of that, the educational industry is being integrated with technology and has faced various challenges in maintaining the documents of academic details for each candidate for extended periods [10].

These challenges happen because due to the large number of data manipulated in the education industry, attracting interest in the development of microservices architectures based on scalability, resilience, and elasticity [11]. Therefore, considering that the use and development of other digital technologies is vital to blocking security threats and adding layers of reliable protection, a powerful opportunity can emerge from utilizing the new blockchain technology [12].

In addition, higher education is considered as an base for the economic, social, and technological development of countries, being related to the formation of human capital and social and technological innovation [9].

Based on that, the importance of stimulating learning emerges due to the loss of enthusiasm generated by the internet. The concern with online content security and learning brings a new technological approach with the use of blockchain, creating safe virtual environments for learning in a motivating way [13].

Similarly, blockchain technology is necessary in the educational arena because it is a significant part of the security process, especially in the verification of documents containing academic details and to provide a reliable solution to avoid any academic fraud [10]. The application of blockchain in the higher education sector is growing, especially in information monitoring carried out through smart applications [14].

Some studies have surfaced with the objective of systematizing the literature in this field of study. Consequently, Castro et al. [5] conducted a study of blockchain and diploma in the Scopus database. In addition, Alzahrani et al. [1] identified articles related to the literature on blockchain and higher education for the transformation of quality 4.0.

Thus far, there is no bibliometric analysis that directly addresses “blockchain technology and higher education”. Based on the context, the study analyzes publications in international literature related to blockchain applications in higher education around the world, conducted using a bibliometric approach. Therefore, the question arises: How blockchain technology is been applied in higher education? This study uses essentially bibliometric analysis to identify opportunities for future investigations and research in the field of digital services for higher education [15,16,17].

## 2. Methodology

### 2.1. Data

The data was compiled from Elsevier’s Scopus online database using documents (Appendix A), published between 2016 and 2021. We have used the boolean method with the terms TITLE-ABS-KEY (blockchain) AND TITLE-ABS-KEY (higher AND education) AND TITLE-ABS-KEY (digital)) AND (LIMIT-TO (LANGUAGE, “English”)) in all literature available till July 2021, which resulted in 61 documents. The following Table 1 summarizes the documents included in this study.

### 2.2. Data Collect

In total, 64 publications were collected, and 3 were excluded due to identified similar publications. All the publications and complementary information were presented in the study. Data were exported in BibTex format [18]. Only conference papers, papers, conference reviews, reviews, and book chapters were included in the search. English language was applied as a filter. Documents’ search was made through bibliographic data (article title, abstract, and keywords) in English and bibliometric studies, mainly using abstract-level data.

### 2.3. Data Synthesis

The publications used in the research was conference papers (*n* = 32), article papers (*n* = 14), conference reviews (*n* = 9), reviews (*n* = 4), and, finally, book chapters (*n* = 2).

### 2.4. Data Analysis

Some Scopus metrics, such as CiteScore, SNIP, and SJR, were used in the analysis tables to understand the data presented [19]. All calculations were performed using Microsoft Excel version 365/2021 (Microsoft Corporation, Washington, WA, USA), to create the graphics. VOSviewer version 1.6.5 software [20] was applied to develop the co-analysis, and Word Art to the cloud analysis and key-words.

## 3. Results

Results are divided into seven sections. The first section characterizes the number of articles by year of publication, namely the published chronological evolution. The second section presents the top ten blockchain publications. The third section presents the top five sources with the largest number of publications, and their score in the field of blockchain. The forth section indicates the number of authors and countries. The fifth section presents authors with the most publications. The sixth section reveals the countries with publications in the field of blockchain in higher education. Finally, the seventh section introduces the most prolific affiliations working with blockchain in higher education.

### 3.1. Articles and Sources

**Figure 1:**
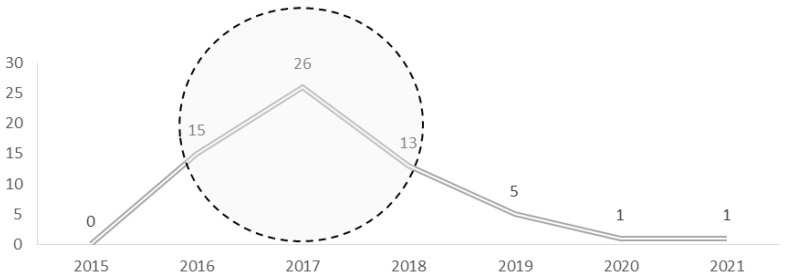
Articles by year of publication.

The publications were searched through the query that included the expression mentioned above, resulting in a total of 61 publications. The annual of evolution can be observed in Figure 1, with the average year of publication being 2018.5 ± 10.16. It was observed that the years 2016 (15 publications), 2017 (26 publications), and 2019 (13 publications) witnessed the major number of publications, while the years 2020 and 2021 saw only one publication each.

In terms of the top of publications, 17 documents presented an average of ±10.64 citations, 7 articles with different numbers presented an average of ±24.14 citations, 3 articles with the same numbers presented 7 citations, 4 articles with the same numbers presented 3 citations, and 3 articles with the same numbers presented 2 citations.

Table 2 presents the top five of blockchain publications.

The five articles with the highest number of citations are:

1. Cheng et al. [21] (53 Citations). In this study, named “Blockchain and smart contract for digital certificate”, the authors show the problem of certificate forgery by proposing a digital certificate system based on blockchain technology. This approach collaborates with Taiwan’s Ministry of Education, ensuring information security for students who receive degrees to enter the job market.
2. Lizcano et al. [8] (38 Citations). In this study, named “Blockchain-based approach to create a model of trust in open and ubiquitous higher education”, the authors evaluate the benefits of blockchain technology and presents a model for transactions based on an academic cryptocurrency. They approach the blockchain to manage content, teaching and competency transactions, assessed by consensus by students, coaches and employers, to eliminate once and for all the “gap” between the academic world and the world of work.
3. Ocheja et al. [22] (27 Citations). In this study, named “Managing lifelong learning records through blockchain,”, the authors presented the overview of the practical implementation of a new platform to track learning achievements, transcripts, and certificates. Discuss the resource requirements and compare the advantages against other similar tools.
4. Swan [23] (26 Citations). In this study, named “Blockchain for Business: Next-Generation Enterprise Artificial Intelligence Systems” the author discusses the Blockchain approach in public and private contexts, considering enterprise deployments and next-generation artificial intelligence systems, notably deep learning blockchains. Other applications can be developed considering global automotive supply chains, healthcare, digital identity accreditation, higher education, and digital collections.
5. Kamišalić et al. [24] (11 Citations). In this study, named “A Preliminary Review of Blockchain-Based Solutions in Higher Education”, the authors presented four types of blockchain initiatives through cases that address different aspects within the educational domain. They consider a preliminary review and analysis of the cases, showing that most follow a student-centered approach.

Table 3 presents sources with the highest number of publications and their score in the field of blockchain (Citescore, SJR, and SNIP). In the first place, we highlight the Journal of Advances in Intelligent Systems and Computing (5 publications), followed by ACM International Conference Proceeding Series and Communications in Computer and Information Science (4 publications each), and, finally, Ceur Workshop Proceedings and Advances in Science Technology and Engineering Systems (2 publications each).

### 3.2. Authors and Countries

Upon analyzing the data from 58 authors of the 61 blockchain documents published in the higher education domain (Table 4), we present the top five main authors. Gouveia and Soares are the authors with the highest number of publications (4 publications each) and Liang and Zhao are the authors with the lowest number of publications (2 publications each).

**Figure 2:**
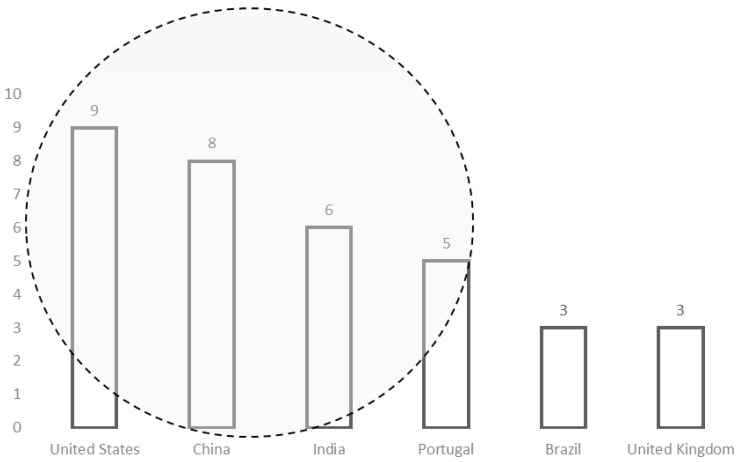
Countries with publications in the field of blockchain in higher education.

The main countries with research competencies in blockchain in higher education are shown in Figure 2. These countries are the USA (9 publications), China (8 publications), India (6 publications), Portugal (3 publications), and the United Kingdom (3 publications).

The most prolific affiliations working with blockchain in higher education with research competencies are shown in Table 5. They are the Universidade Fernando Pessoa (4 publications), the SRM Institute of Science and Technology, University of Central Florida, and Bucharest University of Economic Studies (2 publications each).

### 3.3. Analysis (Co-Citations Analysis, Co-Occurrence Analysis, and Keyword Cloud Analysis)

The initial sample of 64 publications contained 224 citations; however, the sample was reduced to 61 publications after excluding 3 similar references. Based on the analysis of coupling, co-citations, co-occurrence analysis, and cloud, four common reference figures were determined to elaborate the network of connections between the publications and the clustering.

**Figure 3:**
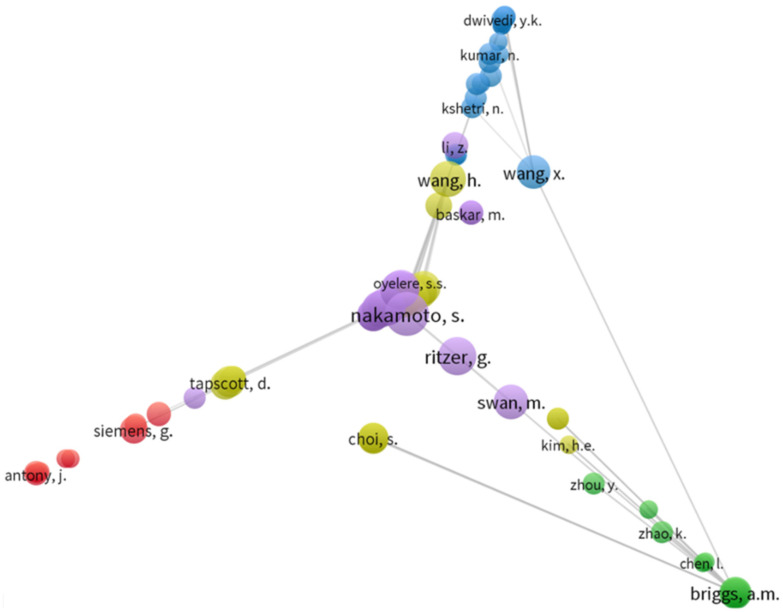
Co-citations analysis.

First, the co-citations analysis among the articles was expressed through Figure 3, based on the common reference data. It is evident that the number of co-citations has been higher for some authors: (1) Nakamoto, S., (2) Wang, H., (3); Wang, X., (4) Tapscott, D., (5) Swan, M., (6) Ritzer, G., (7) Choi, S., (8) Simens, G., and (9) Briggs, A.M.

**Figure 4:**
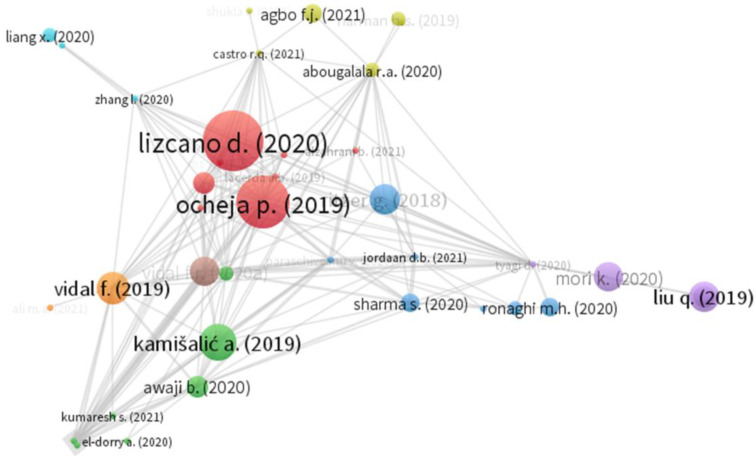
Co-citations analysis.

Second, the coupling analysis was performed to contrast the usual correlation measures among authors. Figure 4 can be large even if there is no direct relationship between the positions. It is evident that the main authors in this analysis were Alzahrani et al. [1] and Ocheja et al. [22] in terms of annual correlation.

**Figure 5:**
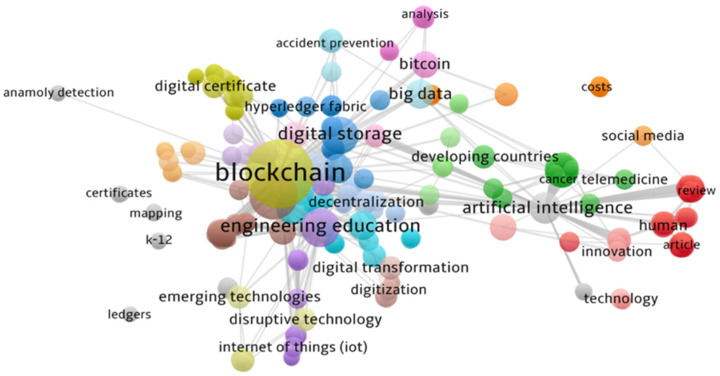
Co-occurrence analysis.

Third, the co-occurrence analysis is simply counting paired data within a collection unit. In this case, paired data “keywords” were used to identify the paired principals as Blockchain and Artificial Intelligence (two complementary words in higher education). Refer to Figure 5.

**Figure 6:**
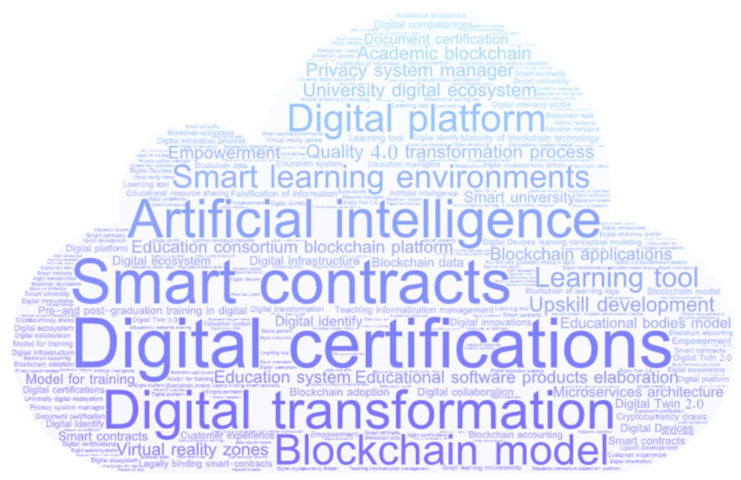
Keyword cloud analysis.

Furthermore, the cloud analysis identified the frequency of key words in publications. It is evident that some key words are more relevant in the publications, such as Artificial Intelligence, Smart Contracts, Digital Transformation, and Blockchain Model. The most important identified word was Digital Certification that has been used in higher education solutions (Figure 6).

### 3.4. Blockchain Technology Applications in the Higher Education Arena

In reference to blockchain, Agbo et al. [25] examined intelligent learning environments, conducting a bibliometric study. In addition, Ali et al. [26] introduced blockchain model to support easy application for certificates. Alzahrani et al. [1] indicated the limited blockchain adoption in support of IES quality. Moreover, Panachev et al. [9] investigated the use of blockchain and game approach in higher education institutions.

In addition, Walcott-Bryant et al. [27] designed a digital healthcare portfolio platform to enable quality and continuity of care. Subsequently, Liang et al. [28] proposed a blockchain network architecture based on the complexity of education scenarios. Furthermore, Kapliienko et al. [29] provided an analysis of data stored in the existing system of diploma verification.

In addition, Sowmiya et al. [14] described the growing popularity of Internet of Things (IoT) systems in monitoring physical attendance. Castro et al. [5] considered that linking blockchain and higher education diplomas can positively impact students around the world. Next, Woods et al. [30] explored the implications for higher education caused by disruptions brought about by changes in the sector.

Subsequently, Ist et al. [31] analyzed the Italian status quo in DH Methods. The Italian Young Medical Doctors Association (Segretariato Italiano Giovani Medici (SIGM)) proposed a web-based survey to assess DH awareness and previous knowledge among young doctors. Investigated areas were big data, omics technology and predictive models, artificial intelligence (AI), internet of things, telemedicine, social media, blockchain, and clinical data storage.

For instance, Jordaan et al. [32] studied the model, LinkLearn, which implements blockchain principles. In addition, Kumaresh et al. [33] described the use of technology to share academic records and student achievement. Furthermore, Tyagi et al. [34] covered technology in various sectors, such as agriculture, social media, banking, education, etc. In addition, Hidrogo et al. [35] developed projects, such as virtual reality zones. Moreover, El-Dorry et al. [36] presented a system for the counterfeiting problem. Similarly, Yue et al. [37] analyzed the influence of blockchain technology on higher education.

Meanwhile, Zhang et al. [38] explored the application of technology to improve the pedagogical information management system in higher education. Chehade et al. [39] discovered a variety of information support consumer education. Interestingly, Ceke et al. [40] explored the possibility of applying intelligence in creating and issuing diplomas.

On top of that, Bolsens et al. [41] described the need to improve the efficiency of organizations with the use of technology. Another point is that Priya et al. [42] indicated that a proposed system model should provide high performance, high efficiency, and low cost, together with the minimum amount of processing time. By detecting anomalies using ML algorithms, the trustworthiness of the documents involved, and transparent transactions are assured. In addition, Abougalala et al. [43] discussed the use of blockchain in smart universities.

In the same way, Awaji et al. [3] examined blockchain applications and summarized the challenges for future studies. Meanwhile, Sharma et al. [44] understood the application of blockchain technology in education. Moreover, Vidal et al. [45] proposed an application for issuing certificates using blockchain technology.

Furthermore, Liang et al. [28] described an educational consortium blockchain-based network. Shukla et al. [46] proposed a model to verify the academic credentials and certificates submitted by students. Additionally, Lizcano et al. [8] proposed a training model to adapt its teaching to the specific needs of students. on the other hand, Zhao et al. [47] proposed a system to identity information in ciphertext form. In addition, Vidal et al. [48] identified, analyzed, and tested the independence, certificate process. Because of that, Pfeiffer et al. [49] presented technologies for storing student data. Another point is provided by Ronaghi et al. [7], assessing the maturity of blockchain technology in agricultural education. Above all, Paraschiveanu et al. [50] featured an article with overviews of the impact of blockchain features.

Indeed, Wishnow et al. [51] conducted research to identify emerging technologies for the oil and gas industry in the coming years. Complementarily, Mori et al. [52] proposed a digital university enrollment system using smart blockchain contracts. Eventually, Ocheja et al. [22] investigated learning records in educational institutions. According to Liu et al. [53], investigated her on the problems of applying blockchain technology. Hou et al. [54] proposed a method for sharing educational resources using blockchain platform. Moreover, Smirnov et al. [55] addressed the dominant role of the consumer in emerging markets using technology.

In addition, Vidal et al. [45] proposed an approach using the blockchain technology at the University Fernando Pessoa. Furthermore, Ricci et al. [56] described that this technology use by individuals could improve awareness and financial education in Ethiopia. Further, Seneviratne et al. [57] presented a high level overview of mobile health (mHealth). In addition, Narman et al. [58] determined the education levels of investors or users who are interested in eight cryptocurrencies by using seven readability techniques. On top of that, Turlacu et al. [59] emphasized that universities should no longer be lagging on technology compared with other sectors.

Although this is not yet explored, new technologies as the next-generation security, the blockchain, cloud, AI conversational interface, and digital credentials can be a leverage for different industries. Above all, Oliveira [60] considered that blockchain technology has emerged as a disruptive trend that can influence business, government, and society in the coming years. In addition, Ma et al. [61] proposed a new model that combines smart contracts.

On the other hand, Fernández et al. [62] observed that the dimension of the cooperative banks influences their perception of the digital transformation in the cooperative banking sector. In this sense, the cooperative banks that affirmed the existence of a wide margin of improvement in the operational scope have a smaller dimension and more seniority than the rest of the sample. Above all, Kamisalic et al. [24] presented different aspects within the educational domain from a case study. Furthermore, Huynh et al. [4] described a proposed model for issuing and verifying digital currency built on blockchain technology.

Similarly, Cheng et al. [21] proposed the digital certificate system based on blockchain technology. In addition, Ritzer et al. [63] reinforced the importance of universities in relation to digital approaches. However, Swan [23] discussed the role of blockchains in next-generation artificial intelligence systems, notably deep learning blockchains. Finally, Neilson et al. [64] provided a set of Bitcoin tutorials for students. The syntheses of authors and blockchain applications are presented in the Table 6.

## 4. Discussion

The objective of our study is to identify the main applications used in the “blockchain technology in higher education” in order to contribute to the literature in this field of study. Thus, we identify three approaches to support this area of interest: blockchain, artificial intelligence, and engineering education. However, with a minimal contribution of literature to the last approach, we focused only on the first and second approaches in our analysis.

Blockchain and Artificial Intelligence are two technologies nowadays accelerating the pace of innovations and promoting significant changes in most diverse sectors, especially in higher education. These results confirmed that the speed of global changes demand a quick adaptation to the new conditions of higher education, creating advances in the application of technologies and innovations in universities [9].

In terms of the blockchain’s contribution to artificial intelligence, features, such as security, efficiency, and energy consumption, can contribute to a decentralized system. With reference to specific contributions, the use and development of other digital technologies is vital to blocking security threats and adding layers of reliable protection, a powerful opportunity can emerge from utilizing the new blockchain technology [12]. These factors can contribute to and improve layers and applications in higher education, thus optimizing usage of blockchain activities.

Regarding the artificial intelligence contribution to blockchain, factors, such as improved user explanation, establishment of clear information chain, and increased machine reliability, can be considered.

Blockchain technology is an advantage over existing ones in that it maintains blockchain record permanence [4], and the number of blockchain-based products is limited in higher education [3].

Therefore, having a chain of blocks helps to efficiently track data, in addition to improving communication between machines. Efficacy can also be perceived as another contributing factor in this relationship, providing more security to the learning data, as well as improving actions and models. As stated by Alzahrani et al. [1], the global evolution has transformed the industrial era into the connected era, with smart products using blockchain applications, especially in contemporary organizations.

The study can relate engineering education with the other two topics as it broadly addresses training in engineering knowledge, in this case, computational. The relationship between blockchain and artificial intelligence is fundamental if we are to bring more value to the innovation process in higher education and to consider future applications for customer needs. Similarly, blockchain technology is necessary in the educational arena because it is a significant part of the security process, especially in the verification of documents containing academic details and to provide a reliable solution to avoid any academic fraud [10].

### Limitations of the Study and Future Line of Research

One limitation includes the need to diversify databases that allow for a better coverage of the blockchain theme in the context of higher education. In the future, accessing international databases with a greater quantity and variety of scientific papers could increase the consistency of the analyses.

Another limitation is the use of analysis tools. It is noticed that tools that are not presented in this study are available in other scientific papers of bibliometric analysis.

The study analyzed blockchain applications in higher education. It could have assessed the impact of blockchain applications as a complement to scientific study.

For further scientific investigation, we propose the operationalization of each of the researched approaches, especially combining the blockchain relationship, artificial intelligence, digital innovation, digital maturity, and customer experience in higher education. For future studies, we suggest to study and intensify research on certain blockchain technologies relevant mainly to higher education and other different sectors:

(a) Investigate how students can have a secure shared data relationship with professors in real time.
(b) A systematic literature review of blockchain enabled applications for scholars and software industries.
(c) Determine the best data approach using blockchain.
(d) Reduce the barrier in the higher education institutes to attract and retain students.
(e) Application of blockchain technologies in the analysis of the digital maturity of health higher education institutions.
(f) Understand the relationship between blockchain technology and digital learners.
(g) Identify whether blockchain technology is at the heart of digital maturity models in the healthcare industry.
(h) Develop a digital maturity model for health sector.
(i) Measure the impact of blockchain technology in learning outcomes (competencies and skills).

## 5. Conclusions

The contributions of this study are to collaborate with the gap of scientific works that offer bibliometric analysis in this domain. This study systematizes the main topics related to the use of blockchain in higher education and presents future investigations, considering the relationship with the topic of digital technologies.

Future implications for theoretical and managerial application can be references in this scientific study, as a possibility that the identified blockchain practices can contribute to higher education institutions in improving (quality and safety) the academic service provided to students.

This general contribution leads to productivity gains and cost reduction, creating possible provisions for the use of money in the higher education institution. This work contributes to the future of higher education institutions in the world, especially in light of the advancement of digitization and the digital transformation of businesses, rethinking the way to serve the student and the way the trust relationship will be with the use of blockchain technology.

## Figures and Tables

**Table 1:** Number of 61 publications included in the study.

Description	Results
Documents	61
Articles	14
Book Chapters	2
Conference Papers	32
Conference Reviews	9
Reviews	4
Sources	2
Keywords Plus	305
Author’s Keywords	178
Period	2016–2021
Average citations per documents	4.23
Principal Authors	42
Documents per Author	1.5
Subject Areas	14
Affiliation	106
Funding Sponsor	18

**Table 2:** Top five of blockchain publications.

	Article	# of Citations
1	Cheng et al. [21]	53
2	Lizcano et al. [8]	38
3	Ocheja et al. [22]	27
4	Swan [23]	26
5	Kamišalić et al. [24]	11

**Table 3:** Top five of sources with the largest number of publications and their score in the field of blockchain.

Source	# Articles	# Citescore	# SJR	# SNIP
Advances in Intelligent Systems and Computing	5	0.9	0.184	0.428
ACM International Conference Proceeding Series	4	1.2	0.182	0.296
Communications in Computer and Information Science	4	0.8	0.16	0.32
Ceur Workshop Proceedings	2	0.8	0.177	0.345
Advances in Science Technology and Engineering Systems	2	0.6	0.139	0.456

**Table 4:** Authors with the most publications in the field of blockchain in higher education.

Authors	Articles
Gouveia, F.	4
Soares, C.	4
Vidal, F.R.	3
Liang, X.	2
Zhao, Q.	2

**Table 5:** The most prolific affiliations working with blockchain in higher education.

Institute	No. of Articles
Universidade Fernando Pessoa	4
SRM Institute of Science and Technology	2
University of Central Florida	2
Bucharest University of Economic Studies	2

**Table 6:** The main applications used in the “blockchain technology in higher education.

Number	Authors	Publication Year	Blockchain Technology Applications
1	(Agbo, Oyelere, Suhonen, and Tukiainen)	2021	Smart learning environments
2	(M A Ali and Bhaya)	2021	Blockchain model
3	(B Alzahrani, Bahaitham, Andejany, and Elshennawy)	2021	Quality 4.0 transformation process
4	(A Panachev, Shcherbitsky, and Medvedev)	2021	Educational software products elaboration.
5	(Walcott-Bryant et al.)	2021	Digital platform
6	(Liang, Zhao, Zhang, Liu, and Zhang)	2021	Education consortium blockchain platform
7	(Kapliienko, Tabunshchyk, Kapliienko, and Wolff)	2021	University digital ecosystem
8	(Sowmiya and Poovammal)	2021	Privacy system manager
9	(R Q Castro and Au-Yong-oliveira)	2021	Diploma certification.
10	(Woods, Doherty, and Stephens)	2021	Upskill development
11	(Casà et al.)	2021	Pre- and post-graduation training in digital
12	[No author name available]/proceedings	2021	-
13	[No author name available]/proceedings	2021	-
14	(Jordaan)	2021	Learning tool
15	(Kumaresh)	2021	Academic blockchain (transparent and secured system for sharing academic records and student’s achievements)
16	(Tyagi, Ghosh, Rana, and Kansal)	2020	Blockchain applications across multiple domains (social medias, education, crypto-currency, information technology and data management)
17	(Hidrogo, Zambrano, Hernandez-de-Menendez, and Morales-Menendez)	2020	Virtual reality zones, collaborative virtual reality, I 360° courses, blockchain for digital credentials, and digital tutors.
18	(El-Dorry et al.)	2020	Document certification
19	(Peng, Yang, and Zhou)	2020	Education system
20	(Zhang, Ma, Ji, and Wang)	2020	Teaching informatization management
21	(Chehade et al.)	2020	Empowerment
22	Ceke, D., Kunosic, S. (Ceke and Kunosic)	2020	Diplomas in education
23	(Bolsens)	2020	-
24	(Priya, Ponnavaikko, and Aantonny)	2020	Verification of certificates
25	(Abougalala, Amasha, Areed, Alkhalaf, and Khairy)	2020	Smart university
26	(B Awaji, Solaiman, and Albshri)	2020	Learning processes (certificate/degree verification, student assessments and exams, credit transfer, data management and admissions)
27	(Sharma and Batth)	2020	Educational bodies model
28	(Vidal, Gouveia, and Soares)	2020	Digital diplomas
29	(Liang et al.)	2020	Microservices architecture (innovation)
30	(Shukla, Indra, Trivedi, Ujjwala, and Monica)	2020	Digital certificates
31	(D Lizcano, Lara, White, and Aljawarneh)	2020	Model for training institution to adapt teaching.
32	(Zhao, Di, and He)	2020	Digital identify
33	(Vidal, Gouveia, and Soares)	2020a	Verifying and sharing certificates.
34	(Pfeiffer, Bezzina, Wernbacher, and Kriglstein)	2020	-
35	(M H Ronaghi)	2020	Maturity of blockchain technology
36	(Paraschiveanu, Richardson, and Voicu-Dorobanțu)	2020	Legally binding smart-contracts; streamline the credentials’ processes and viability and legitimacy of education protection.
37	[No author name available] proceedings	2020	Digital transformation; digital infrastructure; digital ecosystem; digital collaboration; digital competences; blockchain accounting and blockchain adoption.
38	[No author name available] proceedings	2020	Artificial intelligence
39	(Wishnow, Azar, and Rad)	2020	Digital Twin 2.0
40	[No author name available] Proceedings	2020	Blockchain data; smart contracts; learning conceptual modeling and class and object diagrams.
41	(Mori and Miwa)	2020	Falsification of information
42	(Ocheja, Flanagan, Ueda, and Ogata)	2020	Blockchain of learning logs (BOLL) platform
43	(Liu and Zou)	2019	Cooperation innovation of industry, universities, and research institutes.
44	(Hou et al.)	2019	Educational resource sharing
45	(Smirnov, Zakharova, Semenov, Mulendeeva, and Suchkova)	2019	Digital economy profile
46	(F. Vidal, Gouveia, and Soares)	2019	Academic diplomas
47	(Ricci and Mammanco)	2019	Innovative blockchain based system for safety
48	(Seneviratne and Peiris)	2019	Digital health tools, molile.
49	(Narman, Uulu, and Liu)	2019	Cryptocurrency draws
50	(Turlacu, Orzan, Chivu, and Herrezeel)	2019	Customer experience
51	(Ma, Xu, and Xu)	2019	Authenticity of data/smart contracts
52	(Fernández-Torres, Gutiérrez-Fernández, and Palomo-Zurdo)	2019	Digital education process
53	[No author name available] Proceedings	2019	Digital Devices
54	[No author name available] Proceedings	2019	Digital innovations
55	(T T Huynh, Tru Huynh, Pham, and Khoa Ngo)	2018	Education managers
56	(Cheng, Lee, Chi, and Chen)	2018	Smart contract for digital certificate
57	(Ritzer et al.)	2018	Digital transformation
58	[No author name available] proceedings	2018	ERP education; track digital assets of value and digital entrepreneurship.
59	(Swan)	2018	Digital collectibles (cryptokitties); artificial intelligence systems and deep learning algorithms.
60	[No author name available] proceedings	2018	Education services; digital tools and platforms for training programme; digital services and digital transformation.
61	(Neilson, Hara, and Mitchell)	2018	Digital cryptocurrency Bitcoin

## Data Availability

The data sets are available from the corresponding authors on this study.

## Appendix A—List of Publications Review

The productions analyzed (conference proceeding, journal, book series, and book) are: 2018 IEEE International Symposium on Signal Processing and Information Technology, ISSPIT 2018; ACM International Conference Proceeding Series; Advances in Computers; Advances in Intelligent Systems and Computing; AIP Conference Proceedings; Americas Conference on Information Systems 2018; Annali dell’Istituto Superiore di Sanita; CEUR Workshop Proceedings; CIRIEC-Espana Revista de Economia Publica, Social y Cooperativa; Communications in Computer and Information Science; Concurrency Computation; Digital Disruption, AMCIS 2018; eLearning and Software for Education Conference; Eurasip Journal on Wireless Communications and Networking; European Journal of Investigation in Health, Psychology and Education; Industry and Higher Edu-cation; International Conference on Advanced Technologies for Communications; Inter-national Journal on Interactive Design and Manufacturing; Journal of Medical Internet Research; Journal of Physics: Conference Series; Lecture Notes in Business Information Processing; Lecture Notes in Computer Science (including subseries Lecture Notes in Artificial Intelligence and Lecture Notes in Bioinformatics); Lecture Notes in Electrical Engineering; Managing Technology for Inclusive and Sustainable Growth—28th International Conference for the International Association of Management of Technology, IAMOT 2019; Open Review of Educational Research; Proceedings—2019 Chinese Automation Congress, CAC 2019; Proceedings of 4th IEEE International Conference on Ap-plied System Innovation 2018, ICASI 2018; Proceedings—2019 International Conference on Cyber-Enabled Distributed Computing and Knowledge Discovery, CyberC 2019; Proceedings—2020 3rd International Conference on Smart BlockChain, SmartBlock 2020; Proceedings—IEEE Symposium on Computers and Communications; Revolutionizing Tropical Medicine: Point-of-Care Tests, New Imaging Technologies and Digital Health; Proceedings of the 2020 9th International Conference on System Modeling and Advancement in Research Trends, SMART Research and Practice in Technology Enhanced Learning; Smart Learning Environments Sustainability (Switzerland) and Wireless Personal Communications.

## References

[1] Alzahrani B., Bahaitham H., Andejany M., Elshennawy A. (2021). How Ready Is Higher Education for Quality 4.0 Transformation According to the Lens Research Framework?. Sustainability.

[2] Zhang C., Wu C., Wang X. Overview of Blockchain consensus mechanism. Proceedings of the 2020 2nd International Conference on Big Data Engineering.

[3] Awaji B., Solaiman E., Albshri A. Blockchain-based applications in higher education: A systematic mapping study. Proceedings of the 5th International Conference on Information and Education Innovations.

[4] Huynh T.T., Tru Huynh T., Pham D.K., Khoa Ngo A. (2018). Issuing and Verifying Digital Certificates with Blockchain. Int. Conf. Adv. Technol. Commun..

[5] Castro R.Q., Au-Yong-Oliveira M. (2021). Blockchain and higher education diplomas. Eur. J. Investig. Health Psychol. Educ..

[6] Dharmalingam R., Ugail H., Shivasankarappa A.N., Dharmalingam V. (2022). Framework for Digitally Managing Academic Records Using Blockchain Technology. Mobile Computing and Sustainable Informatics.

[7] Ronaghi M.H. (2020). A blockchain maturity model in agricultural supply chain. Inf. Process. Agric..

[8] Lizcano D., Lara J.A., White B., Aljawarneh S. (2020). Blockchain-based approach to create a model of trust in open and ubiquitous higher education. J. Comput. High. Educ..

[9] Panachev A., Shcherbitsky V., Medvedev M.A. (2021). Application of blockchain technologies and game approach in the educational process of universities. AIP Conf. Proc..

[10] Devi O.R. (2015). International Journal of Advanced Trends in Computer Science and Engineering. E3S Web Conf..

[11] Rahman M.A., Abuludin M.S., Yuan L.X., Islam M.S., Asyhari A.T. (2021). EduChain: CIA-compliant block-chain for intelligent cyber defense of microservices in education industry 4.0. IEEE Trans. Ind. Inform..

[12] Ali M.A., Bhaya W.S. (2021). Higher Education’s Certificates Model based on Blockchain Technology. J. Phys. Conf. Ser..

[13] Chang I.F., Chuang Y.H., Chen T.L., Yin Y.P., Liu Y.N., Chen T.S. A Study on the Mechanism of Blockchain Cryptocurrency Implementation: Learning Coin of Campus. Proceedings of the 2020 ACM International Conference on Intelligent Computing and its Emerging Applications.

[14] Sowmiya B., Poovammal E. (2021). A Heuristic K-Anonymity Based Privacy Preserving for Student Management Hyperledger Fabric blockchain. Wirel. Pers. Commun..

[15] Figueiredo R., Ferreira J.J.M. (2020). Spinner Model: Prediction of Propensity to Innovate Based on Knowledge-Intensive Business Services. J. Knowl. Econ..

[16] Figueiredo R., Ferreira J.J.M., Silveira R.G., Villarinho A.T. (2019). Innovation and co-creation in knowledge intensive business services: The Spinner model. Bus. Process Manag. J..

[17] Soliman M., Di Virgilio F., Figueiredo R., Sousa M.J. (2021). The impact of workplace spirituality on lecturers’ attitudes in tourism and hospitality higher education institutions. Tour. Manag. Perspect..

[18] Aria M., Cuccurullo C. (2017). Bibliometrix: An R-tool for comprehensive science mapping analysis. J. Informetr..

[19] Rashid S., Khattak A., Ashiq M., Rehman S.U., Rasool M.R. (2021). Educational landscape of virtual reality in higher education: Bibliometric evidences of publishing patterns and emerging trends. Publications.

[20] Bettencourt S., Costa S., Caeiro S. (2021). Marine litter: A review of educative interventions. Mar. Pollut. Bull..

[21] Cheng J.-C., Lee N.-Y., Chi C., Chen Y.-H. Blockchain and smart contract for digital certificate. Proceedings of the 4th IEEE International Conference on Applied System Innovation 2018 (ICASI).

[22] Ocheja P., Flanagan B., Ueda H., Ogata H. (2019). Managing lifelong learning records through blockchain. Res. Pract. Technol. Enhanc. Learn..

[23] Swan M. (2018). Blockchain for Business: Next-Generation Enterprise Artificial Intelligence Systems. Blockchain Technology: Platforms, Tools and Use Cases.

[24] Kamišalić A., Turkanović M., Mrdović S., Heričko M. (2019). A Preliminary Review of Blockchain-Based Solutions in Higher Education. Commun. Comput. Inf. Sci..

[25] Agbo F.J., Oyelere S.S., Suhonen J., Tukiainen M. (2021). Scientific production and thematic breakthroughs in smart learning environments: A bibliometric analysis. Smart Learn. Environ..

[26] Ali M.A., Bhaya W.S. Higher Education’s Certificates Model based on Blockchain Technology. Proceedings of the Ibn Al-Haitham International Conference for Pure and Applied Sciences (IHICPS).

[27] Walcott-Bryant A., Ogallo W., Remy S.L., Tryon K., Shena W., Bosker-Kibacha M. (2021). Addressing Care Continuity and Quality Challenges in the Management of Hypertension: Case Study of the Private Health Care Sector in Kenya. J. Med. Internet Res..

[28] Liang X., Zhao Q., Zhang Y., Liu H., Zhang Q. (2021). EduChain: A highly available education consortium blockchain platform based on Hyperledger Fabric. Concurr. Comput..

[29] Kapliienko O., Tabunshchyk G., Kapliienko T., Wolff C. (2021). Intellectual Property Assurance Method for Digital University Ecosystem based on Blockchain Technology. CMIS.

[30] Woods R., Doherty O., Stephens S. (2021). Technology driven change in the retail sector: Implications for higher education. Ind. High. Educ..

[31] Ist A., Sanit S. (2021). COVID-19 and digital competencies among young physicians: Are we (really) ready for the new era? A national survey of the Italian Young Medical Doctors Association. Ann. Dell’istituto Super. Sanità.

[32] Jordaan D.B. LinkLearn: Blockchain Technology as a Learning Tool. Advances in Intelligent Systems and Computing. Proceedings of the 13th IMCL Conference.

[33] Kumaresh S. (2021). Academic Blockchain: An Application of Blockchain Technology in Education System. Advances in Intelligent Systems and Computing.

[34] Tyagi D., Ghosh S., Rana A., Kansal V. A comparative analysis of potential factors and impacts that affect blockchain technology in software: Based applications. Proceedings of the 2020 9th International Conference on System Modeling and Advancement in Research Trends (SMART).

[35] Hidrogo I., Zambrano D., Hernandez-de-Menendez M., Morales-Menendez R. (2020). Mostla for engineering education: Part 1 initial results. Int. J. Interact. Des. Manuf..

[36] El-Dorry A., Reda M., El Khalek S.A., El-Din Mohamed S., Mohamed R., Nabil A. Egyptian Universities Digital Certificate Verification Model Using Blockchain. Proceedings of the ACM International Conference Proceeding Series.

[37] Yue P., Xiaofeng Y., Huagang Z. Blockchain Technology and Higher Education: Characteristics, Dilemma and Development Path. Proceedings of the ACM International Conference Proceeding Series.

[38] Zhang L., Ma Z., Ji X., Wang C. Blockchain: Application in the System of Teaching Informatization Management of Higher Education. Proceedings of the 2020 3rd International Conference on Smart BlockChain (SmartBlock).

[39] Chehade M.J., Yadav L., Kopansky-Giles D., Merolli M., Palmer E., Jayatilak A., Salter H. (2020). Innovations to improve access to musculoskeletal care. Best Pract. Res. Clin. Rheumatol..

[40] Ceke D., Kunosic S. Smart contracts as a diploma anti-forgery system in higher education—A pilot project. Proceedings of the 2020 43rd International Convention on Information, Communication and Electronic Technology (MIPRO).

[41] Bolsens I. Scalable system and silicon architectures to Handle the workloads of the Post-Moore era. Proceedings of the International Symposium on Physical Design.

[42] Priya N., Ponnavaikko M., Aantonny R. (2020). Anomaly detection in document verification system using deeplearning in hyperledger. Int. J. Adv. Trends Comput. Sci. Eng..

[43] Abougalala R.A., Amasha M.A., Areed M.F., Alkhalaf S., Khairy D. (2020). Blockchain-enabled smart University: A framework. J. Theor. Appl. Inf. Technol..

[44] Sharma S., Batth R.S. Blockchain Technology for Higher Education Sytem: A Mirror Review. Proceedings of the International Conference on Intelligent Engineering and Management (ICIEM).

[45] Vidal F., Gouveia F., Soares C. Analysis of blockchain technology for higher education. Proceedings of the 2019 International Conference on Cyber-Enabled Distributed Computing and Knowledge Discovery (CyberC).

[46] Shukla A., Indra S., Trivedi T.J., Ujjwala S., Monica C. (2020). Academic credential verification technique using blockchain. Int. J. Adv. Sci. Technol..

[47] Zhao G., Di B., He H. Design and Implementation of the Digital Education Transaction Subject Two-factor Identity Authentication System Based on Blockchain. Proceedings of the International Conference on Advanced Communication Technology (ICACT).

[48] Vidal F.R., Gouveia F., Soares C. (2020). Blockchain application in higher education diploma management and results analysis. Adv. Sci. Technol. Eng. Syst..

[49] Pfeiffer A., Bezzina S., Wernbacher T., Kriglstein S. Blockchain technologies for the validation, verification, authentication and storing of students’ data. Proceedings of the European Conference on e-Learning.

[50] Paraschiveanu V., Richardson G., Voicu-Dorobanțu R. (2020). Education 3.0: Blockchain-backed moocs. Elearning Softw. Educ. Conf..

[51] Wishnow D., Azar H.R., Rad M.P. A Deep Dive into Disruptive Technologies in the Oil and Gas Industry. Proceedings of the Offshore Technology Conference Brasil.

[52] Mori K., Miwa H. Digital University Admission Application System with Study Documents Using Smart Contracts on Blockchain. Proceedings of the INCoS 2019: Advances in Intelligent Systems and Computing.

[53] Liu Q., Zou X. (2019). Research on trust mechanism of cooperation innovation with big data processing based on blockchain. EURASIP J. Wirel. Commun. Netw..

[54] Hou Y., Wang N., Mei G., Xu W., Shao W., Liu Y. Educational Resource Sharing Platform Based on Blockchain Network. Proceedings of the 2019 Chinese Automation Congress (CAC).

[55] Smirnov V.V., Zakharova A.N., Semenov V.L., Mulendeeva A.V., Suchkova A.G. Analysis of the Russian digital economy profile. Proceedings of the ACM International Conference Proceeding Series.

[56] Ricci P., Mammanco V. RemBit: A blockchain based solution for remittances to Ethiopia. Proceedings of the 2019 IEEE Symposium on Computers and Communications (ISCC).

[57] Seneviratne M., Peiris D. (2019). Digital Health in Low and Middle Income Countries. Revolut. Trop. Med..

[58] Narman H.S., Uulu A.D., Liu J. Profile Analysis for Cryptocurrency in Social Media. Proceedings of the 2018 IEEE International Symposium on Signal Processing and Information Technology (ISSPIT).

[59] Turlacu L.-M., Orzan G., Chivu R.-G. Strategic Technologies: Innovation in Higher Education in Romania. Proceedings of the the 15th International Scientific Conference eLearning and Software for Education.

[60] Oliveira T., Thomas M., Espadanal M. (2014). Assessing the determinants of cloud computing adoption: An analysis of the manufacturing and services sectors. Inf. Manag..

[61] Ma X., Xu L., Xu L. Blockchain Retrieval Model Based on Elastic Bloom Filter. Proceedings of the WISA 2019: Web Information Systems and Applications.

[62] Fernández-Torres Y., Gutiérrez-Fernández M., Palomo-Zurdo R. (2019). How do co-operative banks perceive the impact of digital transformation? [¿Cómo percibe la banca cooperativa el impacto de la transformación digital?]. CIRIEC-Espana Rev. Econ. Publica Soc. Coop..

[63] Ritzer G., Jandrić P., Hayes S. (2018). The velvet cage of educational con(pro)sumption. Open Rev. Educ. Res..

[64] Neilson D., Hara S., Mitchell I. Bitcoin forensics: A tutorial. Proceedings of the Global Security, Safety and Sustainability—The Security Challenges of the Connected World.

